# Spontaneous Abortion and a Diet Drug Containing Caffeine and Ephedrine: A Study within the Danish National Birth Cohort

**DOI:** 10.1371/journal.pone.0050372

**Published:** 2012-11-16

**Authors:** Penelope P. Howards, Irva Hertz-Picciotto, Bodil H. Bech, Ellen A. Nohr, Anne-Marie Nybo Andersen, Charles Poole, Jørn Olsen

**Affiliations:** 1 Department of Epidemiology, Rollins School of Public Health, Emory University, Atlanta, Georgia, United States of America; 2 Division of Environmental and Occupational Health, Department of Public Health Sciences, University of California, Davis, California, United States of America; 3 Section for Epidemiology, Department of Public Health, Aarhus University, Aarhus, Denmark; 4 Section of Epidemiology, Department of Public Health, University of Copenhagen, Copenhagen, Denmark; 5 Department of Epidemiology, Gillings School of Public Health, University of North Carolina at Chapel Hill, Chapel Hill, North Carolina, United States of America; Tehran University of Medical Sciences, Iran (Republic of Islamic)

## Abstract

**Background:**

Medications may be consumed periconceptionally before a woman knows she is pregnant. In this study, the authors evaluate the association of a prescription diet drug (Letigen) containing ephedrine (20 mg) and caffeine (200 mg) with spontaneous abortion (SAB) in the Danish National Birth Cohort.

**Methods:**

Women were recruited during their first prenatal visit from 1996–2002. Pre-conception and early pregnancy medication use was reported on the enrollment form, and pregnancy outcome was determined by linking the mother's Civil Registration Number to the Medical Birth Registry and the National Hospital Discharge Register. Of 97,903 eligible pregnancies, 4,443 ended in SAB between 5 and 20 completed gestational weeks, inclusive. Letigen use was reported for 565 pregnancies. Cox regression models accounting for left truncation were fit to estimate the effect of pre-conception and early pregnancy Letigen use on SAB.

**Principal Findings:**

The estimated maternal age-adjusted hazard ratio for SAB was 1.1 (95% confidence interval 0.8–1.6) for any periconceptional Letigen use compared to no periconceptional use.

**Conclusions:**

Although Letigen has high levels of caffeine (the recommended 3 pills/day are approximately equivalent to caffeine from 6 cups of coffee), periconceptional use does not appear to be associated with an appreciably increased hazard of clinically recognized SAB.

## Introduction

Early gestation is a critical time in fetal development when women may be unaware they are pregnant and therefore may use medications, such as diet drugs, that they would not use knowing they were pregnant. Recently, the obesity epidemic in the United States has led the Food and Drug Administration (FDA) to weigh the potential benefits of approving new weight loss drugs against the potential risks, including the risk that reproductive age women may use the drugs before becoming aware of pregnancy. In 2012, the scientific advisory committee to the FDA reversed their 2010 position for one such drug despite concerns that the drug might be associated with an increased risk of oral clefts [1]. In other countries, including Denmark, the prevalence of obesity has also been rising [2], and consequently, reproductive age women may increasingly turn to prescription or over-the-counter weight loss products where available. Letigen (NYCOMED, Denmark) was a prescription diet drug that contained ephedrine and caffeine and was available in Denmark prior to 2002.

**Figure 1:**
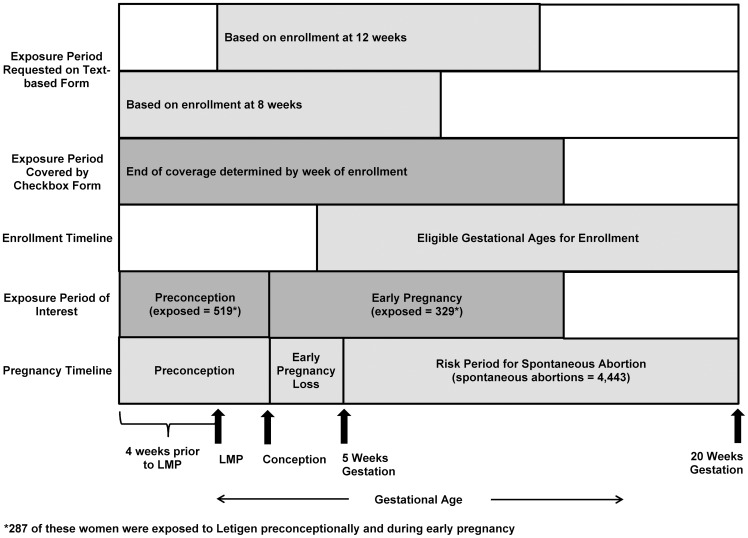
Study timeline. Timelines based on gestational age showing the risk period for the outcome, the exposure period of interest, the timing of enrollment, and the exposure period covered by the enrollment forms used in a Danish National Birth Cohort study of spontaneous abortion and exposure to a diet drug composed of caffeine and ephedrine, 1996–2002.

There has been little research on the effects of ephedrine on pregnancy, but concern over the potential effect of caffeine on spontaneous abortion (SAB) has resulted in a large but inconclusive literature [3]–[26]. After considering this literature, the American College of Obstetricians and Gynecologists published a committee opinion in 2010 stating the current evidence did not suggest that consuming less than 200 mg per day of caffeine during pregnancy increased the risk of SAB, but that the evidence for higher doses of caffeine was still unclear [27]. Similarly, the United Kingdom's Food Standards Agency recommended that pregnant women limit their caffeine consumption to less than 200 mg per day due to concerns about fetal growth restriction and possibly SAB [28]. A number of methodological issues that are difficult to resolve plague studies of caffeine consumption during pregnancy and SAB making it difficult to draw conclusions about the effect of exposure to high doses of caffeine [29]–[33]. Although some of these methodological issues do not apply to pre-pregnancy exposure to caffeine, high exposure to pre-pregnancy caffeine has also been inconsistently associated with SAB with modestly elevated effect estimates in some studies [9], [12], [18], [19] but not others [10], [20], [22]. Most studies of caffeine focused exclusively on caffeine from beverages, including coffee, tea, and cola, that contain other potentially fetotoxic components.

**Table 1:** Summary of counts, fetus-time at risk, and rates per 10,000 for variables stratified on spontaneous abortion (SAB) vs. other pregnancy outcomes for the Danish National Birth Cohort (1996–2002).

	Not SAB		SAB		
	N	Fetus-days*	N	Fetus-days*	Rate of SAB/10,000 fetus-days
**Letigen use** †					
None	92,930	6,547,875	4,408	108,617	6.6
Pre-conception only	219	17,680	13	274	7.2
Both pre-conception and early pregnancy	270	21,311	17	346	7.8
Early pregnancy only	37	2,725	5	71	17.9
Missing timing	4	327	0	0	
**Gestational age at entry (completed weeks)**					
5–8	29,102	2,766,449	2,878	76,695	10.1
9–12	40,260	2,886,894	1,435	30,006	4.9
13–16	18,680	839,147	118	2,465	1.4
17–20	5,418	97,428	12	142	1.2
**Maternal age (years)**					
15–19	987	64,538	40	1,120	6.1
20–24	11,399	818,878	464	11,806	5.6
25–29	38,814	2,797,147	1,609	40,299	5.7
30–34	31,581	2,183,600	1,506	37,775	6.8
35–39	9,756	666,161	683	15,781	10.0
40–46	923	59,594	141	2,527	22.7
**Gravidity**					
No prior pregnancy	30,573	2,215,384	966	25,344	4.3
≥1 prior pregnancy	57,061	3,988,842	2,171	55,864	5.4
Missing	5,826	385,692	1,306	28,100	
**Planned pregnancy**					
Planned	66,705	4,785,563	2,440	63,180	5.0
Partially planned	11,080	765,877	371	9,349	4.8
Not planned	9,859	654,187	328	8,757	4.9
Missing	5,816	384,291	1,304	28,022	
**Infertility treatment**					
Yes	5,700	386,549	167	4,581	4.3
No	81,923	5,817,343	2,972	76,705	5.0
Missing	5,837	386,026	1,304	28,022	
**Prepregnancy body mass index**					
Underweight (<18.5)	3,870	272,001	163	4,507	5.9
Normal (18.5–24.9)	58,493	4,138,880	2,076	52,949	5.0
Overweight (25.0–29.9)	16,707	1,182,334	613	15,805	5.1
Obese (> = 30.0)	7,160	510,755	255	7,114	4.9
Missing	7,230	485,948	1,336	28,933	
**Exercise (hours/week)**					
None	55,769	3,920,465	1,589	43,290	4.0
>0 to <2	17,494	1,241,710	626	16,024	5.0
> = 2 to <4	9,599	697,141	543	13,291	7.6
> = 4 to <7	3,504	254,635	256	5,895	9.8
> = 7	1,271	91,193	124	2,774	13.2
Missing	5,823	384,774	1,305	28,034	
**Smoked during pregnancy (cigarette equivalents/day** § **)**					
None	67,521	4,781,746	2,342	60,576	4.8
>0 to 10	14,741	1,046,138	569	14,975	5.4
>10	5,373	376,952	224	5,656	5.9
Missing	5,825	385,082	1,308	28,101	
**Alcohol during pregnancy (drinks/week)**					
No drinks	48,727	3,507,924	1,560	41,792	4.4
>0 to <4	37,112	2,578,283	1,427	36,548	5.5
> = 4	1,823	120,506	152	2,946	12.3
Missing	5,798	383,205	1,304	28,022	
**Coffee (cups/day)**					
None	48,693	3,507,238	1,477	40,674	4.2
>0 to <4	27,519	1,922,961	1,016	25,636	5.2
4 to <8	8,493	575,907	440	10,171	7.5
> = 8	2,932	198,957	203	4,734	10.0
Missing	5,823	384,855	1,307	28,093	
**Tea (cups/day)**					
None	32,583	2,311,375	1,200	31,184	5.1
>0 to <4	39,427	2,793,264	1,334	34,381	4.7
4 to <8	11,928	842,605	428	11,156	5.0
> = 8	3,699	257,799	176	4,534	6.7
Missing	5,823	384,875	1,305	28,053	
**Cola (liters/week)**					
None	29,967	2,125,366	1,136	30,021	5.3
>0 to <1	42,896	3,026,527	1,310	33,935	4.3
> = 1	14,758	1,051,921	689	17,210	6.4
Missing	5,839	386,104	1,308	28,142	

* Observed fetus days at risk (i.e. time from entry to outcome, censoring, or end of risk period).

† Pre-conception includes the 4 weeks prior to last menstrual period through gestational age 2 completed weeks, early pregnancy includes gestational age 3–13 completed weeks, missing time includes Letigen use during an undefined time.

§ 1 cigarette = 1 cigarette equivalent, 1 cherrot = 2 cigarettes equivalents, 1 cigar = 2 cigarette equivalents, 1 pipe = 1.5 cigarette equivalents.

Letigen was a non-beverage source of high doses of caffeine (200–600 mg/day) that some Danish women used pre-conceptionally and inadvertently early in pregnancy. It was banned in Denmark in 2002 and similar products were banned in the United States in 2004 due to reports of adverse cardiovascular events [34], [35]. We examine whether periconceptional Letigen use is associated with SAB in the Danish National Birth Cohort. Periconceptional Letigen use provides an alternative source of information on exposure to caffeine and SAB with different strengths and weaknesses from studies which focus on caffeine exposure from coffee. Some strengths include that the dose of caffeine is likely more consistent for women using Letigen (600 mg/day if used as directed) compared with women who are exposed to caffeine through coffee, which can be affected by the type of coffee, how the coffee is prepared, as well as changes in personal habits [36]. In addition, nausea is unlikely to lead to mismeasurement of Letigen exposure, but it could affect measurement of caffeine exposure from coffee [10], [30], [32], [33]. Further, exposure to caffeine from Letigen is not confounded by other potentially fetotoxic components of coffee although it could be confounded by ephedrine if ephedrine affects SAB.

**Table 2:** Descriptive statistics by Letigen* exposure status for the Danish National Birth Cohort (1996–2002).

	Non-Letigen*		Letigen*	
	N	(%)	N	(%)
**Gestational age at entry (completed weeks)**				
5–8	31,703	32.6	277	49.0
9–12	41,481	42.6	214	37.9
13–16	18,738	19.3	60	10.6
17–20	5,416	5.6	14	2.5
**Maternal age (years)**				
15–19	1,023	1.1	4	0.7
20–24	11,764	12.1	99	17.5
25–29	40,183	41.3	240	42.5
30–34	32,911	33.8	176	31.2
35–39	10,399	10.7	40	7.1
40–46	1,058	1.1	6	1.1
**Gravidity**				
No prior pregnancy	31,374	32.2	165	29.2
≥1 prior pregnancy	58,874	60.5	358	63.4
Missing	7,090	7.3	42	7.4
**Planned Pregnancy**				
Planned	68,859	70.7	286	50.6
Partially planned	11,336	11.6	115	20.4
Not planned	10,065	10.3	122	21.6
Missing	7,078	7.3	42	7.4
**Infertility treatment**				
Yes	5,852	6.0	15	2.7
No	84,388	86.7	507	89.7
Missing	7,098	7.3	43	7.6
**Prepregnancy body mass index**				
Underweight (<18.5)	4,032	4.1	1	0.2
Normal (18.5–24.9)	60,408	62.1	161	28.5
Overweight (25.0–29.9)	17,109	17.6	211	37.3
Obese (> = 30.0)	7,272	7.5	143	25.3
Missing	8,517	8.7	49	8.7
**Exercise (hours/week)**				
None	57,010	58.6	348	61.6
>0 to <2	18,035	18.5	85	15.0
> = 2 to <4	10,086	10.4	56	9.9
> = 4 to <7	3,739	3.8	21	3.7
> = 7	1,382	1.4	13	2.3
Missing	7,086	7.3	42	7.4
**Smoked during pregnancy (cigarette equivalents/day** † **)**				
None	69,511	71.4	352	62.3
>0 to 10	15,190	15.6	120	21.2
>10	5,546	5.7	51	9.0
Missing	7,091	7.3	42	7.4
**Alcohol during pregnancy (drinks/week)**				
No drinks	49,980	51.3	307	54.3
>0 to <4	38,336	39.4	203	35.9
> = 4	1,962	2.0	13	2.3
Missing	7,060	7.3	42	7.4
**Coffee (cups/day)**				
None	49,842	51.2	328	58.1
>0 to <4	28,410	29.2	125	22.1
4 to <8	8,883	9.1	50	8.8
> = 8	3,115	3.2	20	3.5
Missing	7,088	7.3	42	7.4
**Tea (cups/day)**				
None	33,567	34.5	216	38.2
>0 to <4	40,532	41.6	229	40.5
4 to <8	12,300	12.6	56	9.9
> = 8	3,853	4.0	22	3.9
Missing	7,086	7.3	42	7.4
**Cola (liters/week)**				
None	30,976	31.8	127	22.5
>0 to <1	43,981	45.2	225	39.8
> = 1	15,276	15.7	171	30.3
Missing	7,105	7.3	42	7.4

* Letigen is composed of 20 mg ephedrine and 200 mg caffeine.

† 1 cigarette = 1 cigarette equivalent, 1 cherrot = 2 cigarettes equivalents, 1 cigar = 2 cigarette equivalents, 1 pipe = 1.5 cigarette equivalents.

**Table 3:** Summary statistics for the number of weeks that Letigen* was used at least once among women in the Danish National Birth Cohort (1996–2002).

		Weeks			
Timeframe of use†	N	Mean	SD	Minimum	Maximum
**Total eligible time**	561	6.3	3.3	1	16
**Pre-conception only**	232	4.1	1.9	1	7
**Both pre-conception and early pregnancy**	287	8.7	2.5	2	16
Pre-conception time		6.0	1.9	1	7
Early pregnancy time		2.7	1.5	1	9
**Early pregnancy only**	42	2.3	1.5	1	8

* Letigen is composed of 20 mg ephedrine and 200 mg caffeine.

† Total eligible time includes the 4 weeks prior to last menstrual period through gestational age 13 completed weeks, pre-conception includes the 4 weeks prior to last menstrual period through gestational age 2 completed weeks, early pregnancy includes gestational age 3–13 completed weeks.

Abbreviations: SD, standard deviation.

## Materials and Methods

The Danish National Birth Cohort has been described in detail elsewhere [37]. Briefly, between March 1, 1996 and November 1, 2002, pregnant women across Denmark were provided information about the study during their first prenatal visit. According to a pilot study, approximately 50% of all pregnant women received an invitation to participate, and about 60% of those signed the consent form [37]. The exclusion criteria were not having access to a telephone, not speaking Danish, and not intending to carry the pregnancy to term as of the first prenatal visit. A total of 101,051 pregnancies were enrolled.

**Table 4:** Unadjusted and age-adjusted hazard ratios and 95% confidence intervals for the association between Letigen* use and spontaneous abortion (SAB) in the Danish National Birth Cohort (1996–2002).

			Unadjusted		Age adjusted†	
Timeframe of exposure§	SAB	Fetus-days$	HR	(95% CI)	HR	(95% CI)
**None**	4,408	6,656,492	1.0	ref	1.0	ref
**Pre-conception only**	13	17,954	1.0	0.6; 1.6	1.0	0.6; 1.7
**Both pre-conception and early pregnancy**	17	21,657	1.0	0.6; 1.7	1.1	0.7; 1.7
**Early pregnancy only**	5	2,796	2.6	1.1; 6.5	2.7	1.1; 6.6

* Letigen is composed of 20 mg ephedrine and 200 mg caffeine.

† Adjusted for maternal age using the following categories: 15–19, 20–24, 30–34, 35–39, 40–46 with 25–29 as the referent.

§ Pre-conception includes the 4 weeks prior to last menstrual period through gestational age 2 completed weeks; early pregnancy includes gestational age 3–13 completed weeks.

$Observed fetus days at risk (i.e. time from entry to outcome, censoring, or end of risk period).

Abbreviations: HR, hazard ratio; CI, confidence interval.

The Danish National Birth Cohort was approved by the Danish Scientific Ethics Committee, and this specific study was approved by the Danish Data Protection Board. All women participating in the cohort provided written informed consent.

### Pregnancy Outcome

Pregnancy outcome was assessed from Danish national registries, which can be linked using the unique identifier, the Civil Registration Number. The Medical Birth Registry and the Civil Registration System were used to obtain data on live and still births. Other pregnancy outcomes were identified in the National Hospital Discharge Register, and emigration prior to the end of pregnancy was determined from the Civil Registration System. Less than one percent of the study pregnancies could not be linked to registry data; in these cases, outcome information from the interviews was used instead [4]. We excluded 34 pregnancies with unknown outcomes and 142 ectopic and molar pregnancies.

We defined SAB as an involuntary intrauterine loss between gestational days 35–146, inclusive (i.e. 5–20 completed weeks) (Figure 1). Primarily, we based gestational age on the National Hospital Discharge Register, which contained an estimate of gestational age that was usually based on a sonogram. However, for 391 pregnancies, we used the last menstrual period (LMP) date reported on the enrollment form because it seemed reasonable and the date from the National Hospital Discharge Register was missing or seemed incorrect. We excluded 124 pregnancies with missing or erroneous gestational ages, 586 pregnancies enrolled after the outcome, 172 pregnancies allegedly enrolled prior to 28 days post-LMP, and 2,090 pregnancies enrolled after 146 days gestation (i.e. no observed time at risk for SAB), yielding a total of 97,903 pregnancies. Eligible multiple births (n = 2,045) were treated as a single event; we treated the one eligible pregnancy with discrepant outcomes as a SAB.

### Exposure Assessment

The exposure of interest was periconceptional use of Letigen. Each pill contained 20 mg of ephedrine and 200 mg of caffeine; the recommended dose was three pills per day [38]. Thus, women using Letigen as directed ingested caffeine approximately equivalent to drinking five to six cups of coffee a day [39], as well as ephedrine, and possibly caffeine from other sources. Letigen use was reported on the enrollment form prior to the pregnancy outcome. A pregnancy was defined as exposed if any Letigen use was reported on the enrollment form during the four weeks prior to the woman's LMP through 13 completed weeks post-LMP (n = 565). Twenty-two pregnancies were classified as unexposed despite reported Letigen use because the use predated the periconceptional period. Four women did not report when they used Letigen; we assumed they were exposed. We also defined pre-conception Letigen use as occurring four weeks prior to LMP through two completed gestational weeks, and early pregnancy use as occurring from three to 13 completed gestational weeks (Figure 1).

Classification of exposure status was complicated by the fact that there were two versions of the enrollment form. On the original form, used for 65% of the pregnancies, medication type and timing of use were reported in text fields for the three months prior to filling out the form (Figure 1). On the revised form, the participant listed medications consumed and marked boxes to indicate weeks when the medication was used. The boxes covered the four weeks prior to the participant's LMP through enrollment or 13 completed weeks post-LMP, whichever came first (Figure 1).

Four factors affected Letigen exposure assignment. First, women enrolled at different times in their pregnancies and therefore reported Letigen use through different gestational ages (Figure 1). We assumed that no one took Letigen after they knew they were pregnant. Given that all women knew they were pregnant at enrollment, this translated into assuming that no one took Letigen after enrollment. This assumption seems reasonable because almost all women specifically stated that they stopped taking Letigen when they became aware of their pregnancy or clearly reported cessation of use prior to enrollment. This is also consistent with a recent United States study of birth defects and retrospectively-reported weight loss products (including over-the-counter products) that reported exposure dropping from over 1% prior to pregnancy to 0.2% by the third month of pregnancy [40].

For each version of the enrollment form, the mean gestational age at entry was ten (standard deviation (SD) three) weeks. However, the two forms referred to different timeframes (Figure 1). The exposure period of interest was covered by the checkbox enrollment form and the text-based form for women enrolling by eight weeks. However, women who used the text-based form and enrolled after eight completed weeks were asked only about part of this timeframe. In practice, Letigen users who completed the text-based form and enrolled late tended to report medication usage covering the entire timeframe of interest (i.e. usage more than three months prior to enrollment). However, women who used the text-based enrollment form and did not report Letigen use might be mistakenly classified as unexposed if they used Letigen pre-conceptionally and enrolled late. We examined this issue analytically by performing sub-analyses where we excluded late enrollers or stratified on the version of the form used.

The third issue was that the timing of Letigen use was often reported vaguely on the text-based form. We reviewed each text field individually and developed rules regarding the timing of exposure for commonly used phrases (e.g., six days in early September was assumed to be the middle six days in the first ten days of the month). Because we assessed broad exposure windows, most women were clearly exposed or unexposed periconceptionally, despite nonspecific responses.

The final challenge was that women reported Letigen use relative to self-reported LMP, which was not always consistent with the estimated LMP based on the National Hospital Discharge Register. For 87% of the women, the dates were within a week of each other (30% were identical) so reports pertained to the relevant timeframe despite minor date discrepancies. Of the 1,452 pregnancies where LMP dates differed by more than four completed weeks and the National Hospital Discharge Register date was determined to be the best estimate, 11 women reported using Letigen. Their exposure status did not change regardless of the gestational age definition used. Some of the 1,441 women who were classified as unexposed might have been exposed if they had reported on the relevant timeframe only, but the number is likely to be small. We addressed this uncertainty through a sensitivity analysis excluding pregnancies for which gestational age was based on the National Hospital Discharge Register and for which the self-reported and Register-based LMPs differed by more than four completed weeks.

### Other Variables

Maternal age at LMP was calculated using birth date from the Civil Registration System. Information on all other covariates was collected through telephone interviews. Of the 97,903 pregnancies in this study, 7,094 were never interviewed. Of the women contacted for the first pregnancy interview, 3,144 were no longer pregnant and therefore were not interviewed at that time. The mean gestational age at interview for women who were still pregnant was 16 completed weeks (range 5–37 weeks). Approximately 80% of the women who were no longer pregnant agreed to participate in an alternative interview, which for our purposes, was comparable to the pregnancy interview.

### Statistical Analyses

We fit Cox regression models accounting for left truncation to estimate the effect of Letigen on SAB. We used gestational age in days with entry into observation defined as enrollment in the study. We employed the robust Lin and Wei confidence interval option in SAS 9.2 (Cary, North Carolina) to account for the fact that some women (n = 8,099) contributed more than one pregnancy to the study. Pregnancies that ended in an induced abortion (n = 425) and where the mother died (n = 3) or emigrated (n = 48) prior to 20 completed weeks gestation were censored.

We considered the following potential confounders: maternal age, pre-pregnancy body mass index (BMI), exercise, smoking, alcohol consumption, and consumption of caffeine from beverages. Although these factors could conceivably be confounders or proxies for confounders, none substantially changed the effect-measure estimates for Letigen separately or as a group (<3% change in estimate). Given that a higher proportion of women with SABs did not have interviews compared with women with other pregnancy outcomes (29% vs. 6%) and the fact that none of the other covariates appeared to confound the effect-measure estimate considerably, we opted to report the results for the model adjusted for maternal age only. This allowed us to include all eligible pregnancies.

## Results

Table 1 provides counts and observed fetus-time at risk for the study population by outcome. As expected, older women were more likely to have a pregnancy loss, and as previously published, SAB was associated with higher coffee consumption during pregnancy [4]. Letigen use was strongly associated with higher prepregnancy BMI (Table 2). In addition, Letigen users tended to drink more cola than non-users, but the two groups were similar on all other factors including reported coffee consumption and tea consumption. Among Letigen users, the average number of gestational weeks with any Letigen use was 6 (SD 3) during the exposure period interest (Table 3). Most Letigen use was reported during the pre-conception period rather than early pregnancy.

The maternal age-adjusted hazard ratio (HR) for any Letigen use compared to no use was 1.1 (0.8–1.6). Table 4 shows unadjusted and age-adjusted HRs from models examining Letigen use during different timeframes. Only women who used Letigen during early pregnancy exclusively had an elevated hazard (adjusted HR 2.7; 1.6–6.6), but there were only five SABs exposed to Letigen during this timeframe.

We conducted additional analyses to examine the sensitivity of our results to possible misclassification of Letigen use. First, we excluded women who used the text-based form and enrolled after eight weeks gestation; the age-adjusted HR did not change substantially from that of the complete study population (1.2; 0.8–1.7). Excluding all women who enrolled after eight weeks gestation, regardless of which enrollment form was used, also did not change the estimate (1.1; 0.8–1.6). Limiting to pregnancies dated by self-reported LMP (i.e. LMP from the enrollment form where medication use was reported) or where the Register estimated LMP differed from the self-reported LMP by fewer than five weeks yielded an age-adjusted HR of 1.2 (0.9–1.7). Finally, restricting our analysis to nulligravida women yielded in an age-adjusted HR of 1.1 (0.5–2.4).

## Discussion

Periconceptional Letigen use is not appreciably associated with SAB in this study. Although pre-pregnancy Letigen use showed little or no association with SAB, early pregnancy use was associated with an elevated HR. However, the elevated HR was based on only five exposed SABs. If Letigen were causally related to SAB when taken in early pregnancy, it would be expected to be harmful for any exposed pregnancy, whether or not the woman also took it prior to conception. However, women exposed during both periods showed no elevated risk. Further, the effect measure estimate for Letigen use during early pregnancy only may be confounded. Although adjusting for measured potential confounders did not change the estimate meaningfully, it is possible that women who began using Letigen after conception may have been more likely to participate in unmeasured behaviors that increase the hazard of SAB.

Some assumptions were made to determine exposure status during the timeframe of interest; to the degree that we could test these assumptions, the overall effect-measure estimates appeared robust. We accounted for left truncation analytically. However, this approach assumes that the pregnancies under observation at a given gestational age are representative of all pregnancies at that gestational age. Bias could be introduced if this was not the case, especially if entry was associated with the exposure [41]. Further, gestational age may be less accurate for SABs because live births are more likely to have a sonogram. However, previous work suggests bias due to differential accuracy in gestational age by outcome is likely to be small given realistic assumptions about the magnitude of these differences [42].

Letigen was composed of ephedrine and caffeine. To our knowledge, no prior studies looked at weight loss products in relation to SAB. A National Birth Defects Prevention Study reported that periconceptional use of weight loss products containing ephedra (a botanical source of the alkaloid ephedrine) was associated with increased risk for some birth defects although the effect-measure estimates were imprecise [40]. While the relation between diet drugs and SAB has not been studied, an extensive literature addresses caffeine and SAB [3]–[26]. Opinions published in both the United States and the United Kingdom could not reach consensus regarding the relation between consumption of high doses of caffeine during pregnancy and SAB, but both recommended women limit their consumption to less than 200 mg per day [27], [28].

High coffee consumption (greater than 4 cups per day) during pregnancy is associated with late SAB in the Danish National Birth Cohort [4], which contrasts with the results for periconceptional Letigen consumption. The results may differ for several reasons. First, the study of coffee and SAB only included late losses because pregnancies interviewed after the outcome were excluded to avoid recall bias. In contrast, earlier losses were included for periconceptional Letigen use, which was reported at enrollment, precluding recall bias. Second, although both coffee and Letigen contain caffeine, coffee also contains other substances that may affect SAB. Additionally, caffeine levels from coffee were likely based on coffee consumption at the time of the interview, which probably represented average daily exposure after becoming aware of pregnancy. In contrast, while the dose of caffeine may be consistent for women taking Letigen (ranging from 200 to 600 mg of caffeine depending on the number of pills consumed), the timing of use during early pregnancy may have been brief or sporadic. Letigen use was reported for an average of less than three weeks during the exposure period of interest. It is possible that regular exposure to high doses of caffeine affects SAB but sporadic exposure to high doses does not.

One concern in studies of caffeine consumption during pregnancy is that pregnancy induced nausea can lead to food and beverage aversions, while lack of nausea is strongly associated with an increased risk of SAB. As a result, observed associations between caffeine and SAB could be spurious [10], [30], [32], [33]. An advantage of assessing pre-pregnancy caffeine consumption is that the exposure predates the onset of nausea so any observed association is not a result of nausea's effect on caffeine consumption. However, pre-conception caffeine exposure cannot be generalized to caffeine exposure during pregnancy because effects may differ during different developmental periods. In addition, pre-conception caffeine exposure may have an effect on early pregnancy loss (<5 completed weeks), but not on clinically recognized SAB.

Women tended to cease using Letigen once they became aware of their pregnancy. Therefore, there was limited early pregnancy exposure in this study, but Letigen users were exposed to caffeine for longer periods prior to conception. Researchers examining pre-pregnancy (beverage) caffeine consumption and SAB [9], [10], [12], [14], [18]–[20], [22] reported inconsistent results even for high levels of caffeine. However, the effect-measure estimates for high caffeine intake prior to pregnancy are smaller than estimates for high caffeine intake during pregnancy in studies that looked at both periods. We also found a stronger association between SAB and Letigen use during early pregnancy compared to pre-conceptional use although relatively few women consumed Letigen in early pregnancy exclusively. The association between SAB and Letigen use during both periods was essentially null.

We did not have adequate data to evaluate whether caffeine from other medications might confound the association between Letigen and SAB. Some painkillers and migraine medications that contained caffeine were available during the study period. These types of drugs were likely used sporadically and had much lower caffeine content (50–100 mg/pill) than Letigen (200 mg/pill). Therefore, it seems likely that few study participants were regularly exposed to high doses of caffeine from other medications.

We evaluated whether caffeine from beverages could confound this study. Although women were asked about coffee, tea, and cola consumption, the questions were not anchored to a specific time period. We considered whether reported caffeine intake from beverages could approximate periconceptional caffeine consumption as well as whether caffeine from beverages might be an intermediate between Letigen and SAB. If the latter were true, adjustment for beverage caffeine would be inappropriate. Only cola consumption appeared to be associated with Letigen use in our data. High prior cola consumption could contribute to obesity, which could lead to Letigen use, or alternatively, obesity could lead to both Letigen use and diet cola consumption. In both of these scenarios cola could be a confounder if caffeine from cola caused SAB. Adjustment for cola consumption did not change the results appreciably (1.0; 0.7–1.5) although residual confounding due to imprecise measurement of cola consumption is possible.

In the Danish National Birth Cohort, periconceptional use of a diet pill containing ephedrine and caffeine does not appear to be appreciably associated with clinically recognized SAB.
